# Synthetic MRI improves radiomics‐based glioblastoma survival prediction

**DOI:** 10.1002/nbm.4754

**Published:** 2022-05-21

**Authors:** Elisa Moya‐Sáez, Rafael Navarro‐González, Santiago Cepeda, Ángel Pérez‐Núñez, Rodrigo de Luis‐García, Santiago Aja‐Fernández, Carlos Alberola‐López

**Affiliations:** ^1^ Laboratorio de Procesado de Imagen Universidad de Valladolid Valladolid; ^2^ Departamento de Neurocirugía Hospital Universitario Río Hortega Valladolid Spain; ^3^ Departamento de Neurocirugía Hospital Universitario 12 de Octubre Madrid Spain

**Keywords:** glioblastoma, synthetic MRI, radiomics, survival prediction

## Abstract

Glioblastoma is an aggressive and fast‐growing brain tumor with poor prognosis. Predicting the expected survival of patients with glioblastoma is a key task for efficient treatment and surgery planning. Survival predictions could be enhanced by means of a radiomic system. However, these systems demand high numbers of multicontrast images, the acquisitions of which are time consuming, giving rise to patient discomfort and low healthcare system efficiency. Synthetic MRI could favor deployment of radiomic systems in the clinic by allowing practitioners not only to reduce acquisition time, but also to retrospectively complete databases or to replace artifacted images. In this work we analyze the replacement of an actually acquired MR weighted image by a synthesized version to predict survival of glioblastoma patients with a radiomic system. Each synthesized version was realistically generated from two acquired images with a deep learning synthetic MRI approach based on a convolutional neural network. Specifically, two weighted images were considered for the replacement one at a time, a T2w and a FLAIR, which were synthesized from the pairs T1w and FLAIR, and T1w and T2w, respectively. Furthermore, a radiomic system for survival prediction, which can classify patients into two groups (survival >480 days and ≤ 480 days), was built. Results show that the radiomic system fed with the synthesized image achieves similar performance compared with using the acquired one, and better performance than a model that does not include this image. Hence, our results confirm that synthetic MRI does add to glioblastoma survival prediction within a radiomics‐based approach.

## Graphical abstract

Glioblastoma is a common brain tumor, with poor prognosis. Radiomic systems (RSs) may improve patient care as an aid to predict survival and personalize treatments. Synthetic MRI favors deployment of RSs by reducing acquisition time and curating databases. Whether an RS can reliably work on synthesized images needs verification. We found that an RS fed with a set of images of which one is synthesized performs similarly to one fed with acquired images, and better than one that ignores the synthesized image.

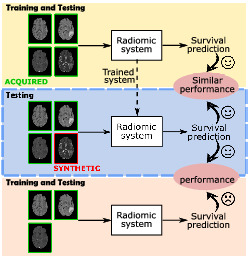

**Abbreviations**
AUC: area under the curve
BraTS2020: multimodal brain tumor segmentation
CNN: convolutional neural network
ED: edema
ET: enhancing tumor
ICC: intra‐class correlation coefficient
IQR: interquartile range
IR‐SE: inversion recovery spin echo
KPS: Karnofsky performance status
LR: logistic regression
MAE: mean absolute error
mRMR: maximum relevance–minimum redundancy
MSE: mean squared error
NET: non‐enhancing tumor
PD: proton density
PET: positron emission tomography
PSNR: peak signal to noise ratio
ROI: region of interest
RS: radiomic system
SE: spin echo
SP: survival prediction
SSIM: structural similarity index
SVM: support vector machine
TC: tumor core
TCIA: The Cancer Imaging Archive
T1w: $T_{1}$ weighted
T1w‐c: T1w post‐contrast
T2w: $T_{2}$ weighted
WT: whole tumor
XGB: extreme gradient boosting

## 1 INTRODUCTION

Glioblastoma is an aggressive and fast‐growing brain tumor, which comprises the most common kind of malignant brain tumor.^1^ Recently, several advances have been achieved in precision oncology and immunotherapy.^2^ However, the overall survival remains poor, with approximately 40% survival in the first year after diagnosis and 17% in the second year.^3^ Thus, survival prediction (SP) in glioblastoma prognosis is a key task for efficient treatment and surgery planning.

Factors commonly used for SPs are age, sex, extent of resection, or Karnofsky performance status (KPS)^4^—a functional status metric for activities of daily living.^5^ Although these factors have shown ability to predict survival rates, their results are still poor and nowadays there is no clinical standard based on these metrics.^6^ Moreover, some of these factors are subjective (e.g., KPS), so clinicians are forced to rely on their previous experience and intuition. Therefore, a quantitative predictive tool is still an unmet need.

Radiomics and medical images are two major actors to achieve such a quantitative predictive tool. Radiomics^7^ is a discipline that consists in the extraction of a large number of quantitative features from the images, the selection of a subset of them according to some quality criteria, and the design of an inference engine that will carry out the clinical predictions with the remaining features as inputs. Radiomics has been an useful technique, pioneering in the oncology field, for quantitative decision making in diagnosis, prognosis, and therapeutic response.^8^ In particular, radiomic systems (RSs) for SP in glioblastoma could enhance patient management by treatment personalization, giving rise to better outcomes.^9^

Regarding the second actor, MRI is a widely used medical imaging technique in different fields—and, particularly, in neuro‐oncology—for non‐invasive diagnosis and evaluation of disease progression. An MRI scan protocol typically consists of several pulse sequences that provide images with different contrasts, each of which is referred to as a weighted image, and they are collectively referred to as multicontrast images. Each image weighting provides complementary information for diagnosis, since different tissue properties may be more clearly visible in each of them.^10^ The use of multicontrast images is also crucial for radiomics. However, multicontrast acquisitions are time consuming, which results in patient discomfort and artifact‐prone protocols. For protocol shortening, the emerging field of synthetic MRI lends itself to become a cornerstone.

Synthetic MRI comprises methodologies pursued to computationally synthesize realistic MR images from a set of actually acquired images. This discipline has been boosted by deep learning techniques. Synthetic MRI has many potential applications as for image database management, such as retrospective completion of databases with missing weighted images or replacement of artifacted images. Additional applications such as data harmonization^11^ or data augmentation^12^, ^13^ for segmentation or classification algorithms have recently been proposed. As for multicontrast acquisitions, synthetic MRI may be applied to replace some acquisitions with their synthesized versions, leading to MR protocol shortening, patient wellbeing and higher efficiency. To make this possible, synthesized MR images should be of sufficient quality, and this is usually assessed both visually and with specific image quality assessment metrics. However, it is also essential to measure how these synthesized images will perform with quantitative algorithms.^14^

In this work we propose and quantify the application of synthetic MRI to improve a radiomics approach for SP in glioblastoma; both the RS and the image synthesis method are original and the details of their design are fully described. Our purpose is to show that an RS that incorporates an input channel fed by a synthesized image (a) behaves similarly to this system when it is fed with an acquired image and (b) undoubtedly outperforms an RS that does not have this channel. Hence, we validate an MR protocol shortening procedure by means of a glioblastoma SP radiomics‐based application. Two weighted images are considered for the synthesis, namely, FLAIR and $T_{2}$ weighted (T2w). We synthesize these images by means of an improved version of our previous deep learning approach for relaxometry maps synthesis.^15^ Our results allow us to state that synthetic MRI does add to glioblastoma SP within a radiomics‐based approach.

### 1.1 Related work

The quality assessment of synthesized images generally focuses on their usage in qualitative applications, and it remains to be verified whether quantitative algorithms can reliably work with these synthesized images. In particular, to the best of our knowledge, the performance of synthesized images with a clinical endpoint in radiomics applications has not been thoroughly tested.

Recent works make use of synthesized images to improve subsequent quantitative image analysis algorithms. In Reference ^16^ an MRI synthesis method was presented, and the quality of a tumor segmentation algorithm was used as a benchmark to compare different synthesis procedures. Furthermore, in Reference ^17^ a generative adversarial network was trained for MRI synthesis and was then used for data augmentation in Parkinson's disease classification. The usage of synthesized data showed an improvement in the classification performance. Moreover, Sikka et al^18^ proposed the synthesis of positron emission tomography (PET) images from MRI. The synthesized PET images were then included in an Alzheimer's disease classification task, for which a relevant accuracy improvement was also shown.

Regarding the application of synthesized images in glioblastoma, we are aware of two contributions^11^, ^19^ that have used synthesized images as input. In Reference ^11^ image synthesis is used to fill missing contrasts in MRI glioma databases. The overall performance is measured by means of tumor segmentation accuracy, as well as by the merit figures of two RSs, one for tumor grading and the other one for isocitrate dehydrogenase‐1 (IDH1) status prediction. Completion of the databases with synthesized images produced an improvement in the results of these tasks. However, synthesized images are apparently used for training and testing of the RSs, which implies a coupling between these systems and the corresponding synthesis method.

Islam et al^19^ proposed a radiogenomics system for overall SP in glioblastoma using an MRI synthesis method to complete the missing images in the dataset. Tumor segmentation and overall SP were tested with the synthesized data. Nevertheless, the classifiers themselves were trained on the synthesized images; hence, this methodology is a data augmentation procedure and benefits in SP may arise not only from the quality of the synthesized images, but also from the fact that more data are used, as pursued in data augmentation. Hence, this methodology does not provide sufficient evidence that acquired images can be replaced by their synthesized versions in a quantitative radiomic application, since two effects are coupled. Furthermore, the authors predominantly use morphological characteristics rather than intensity or texture‐based features, although the latter are more directly related to the intensity image values so as to measure the impact of the synthesized images. The aforementioned limitations of Reference ^19^ are overcome in the present work, since our RS is solely trained with acquired images while synthesized images are exclusively used for testing. In addition, intensity and texture features are also considered in the RS that we propose.

## 2 MATERIALS AND METHODS

**FIGURE 1:**
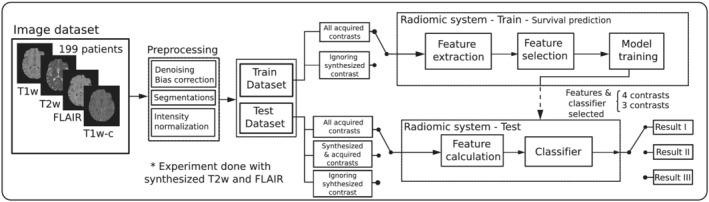
Workflow of the proposed approach. Initially, patients are divided into training (175 patients) and testing (24 patients) sets. The preprocessing pipeline segments tumors and normalizes the contrast intensity. Features are retrieved from the segmented regions of interest (ROIs). After feature selection, relevant features are retained. Five machine learning models for SP were examined. Three different experiment configurations (referred to throughout the text as Experiments/Results I, II, and III) were defined for comparative performance assessment

In this work we propose the application of synthetic MRI to improve an RS for SP in glioblastoma. Following the flow provided in Figure 1, Section 2.1 describes the datasets used in this work and Section 2.2 the data preprocessing stage. Feature extraction and selection as well as classifier training is described in Section 2.3. Section 2.4 describes the procedure for the synthesis of weighted images, which enter the RS exclusively at test time through the feature calculation block. Finally, Section 2.5 describes the three experiments we have carried out to obtain Results I, II, and III listed in Figure 1.

### 2.1 Datasets and MR image acquisition

Four different datasets of glioblastoma patients were used in this work. Two of them are publicly available, namely, the BraTS2020 (multimodal brain tumor segmentation)^20^ Challenge dataset, and the datasets reachable through TCIA (The Cancer Imaging Archive)^21^—which, in turn, consist of thee sources, namely, the Ivy Glioblastoma Altas Project (Ivy‐GAP), the Clinical Proteomic Tumor Analysis Consortium Glioblastoma Multiforme (CPTAC) and The Cancer Genome Atlas Glioblastoma Multiforme (TCGA). The other two (Dataset22 and Dataset24), are private datasets acquired in Hospital Universitario Río Hortega, Valladolid, Spain, and Hospital Universitario 12 de Octubre, Madrid, Spain, respectively. Details of the datasets can be found in Table 1. From all the datasets, we only included those patients (199 patients in total) in whom gross total resection (100% of the enhancing tumor (ET) volume) or near‐total resection (>95% of the ET volume) could be performed. The cases selected from the public datasets are referenced in Supporting Information Table S1.

**TABLE 1:** Datasets used in this work. BraTS2020 and TCIA are public datasets, whereas Dataset22 and Dataset24 are private datasets. The number of patients in each dataset is denoted by n. Age is shown as mean ± standard deviation. Survival is defined as the time in days from diagnosis to death (censored = 0) or to the last date the patient was known to be alive (censored = 1). The percentages of patients with survival less than 16 months (survival < 16 M) for the different datasets are also displayed (16 months or, equivalently, 480 days)

Dataset	n	Age	Survival (IQR)	% Censored = 1	Survival < 16 M
BraTS2020	119	62 ± 12	374 (364)	0%	65.6 %
TCIA	34	60 ± 10	521 (482)	5.9 %	58.8 %
Dataset22	22	65 ± 10	451 (307)	22.7 %	59.1 %
Dataset24	24	57 ± 13	552 (218)	29.2 %	54.2 %
Whole datset	199	60 ± 11	447 (346)	7.0 %	62.3 %

For each patient, four MR structural weighted images— $T_{1}$ weighted (T1w), T2w, FLAIR, and T1w post‐contrast (T1w‐c)—were available. All the acquisitions of the private datasets were performed with IRB approval and informed written consent. See Table 2 for details of the acquisition parameters.

**TABLE 2:** All MRI sessions are composed of four structural weighted images, namely, a T1w, a T2w, a FLAIR, and a T1w‐c. Details of the scanner and the acquisition parameters are provided if available. The acquisition parameters are echo time ( $T_{E}$), repetition time ( $T_{R}$), and inversion time ( $T_{I}$)

Dataset	Scanner	T1w	T2w	FLAIR	T1w‐c
BraTS2020	19 institutions	NA	NA	NA	NA
TCIA	8 institutions	$T_{E}$ = 2.75–19 ms	$T_{E}$ = 15–120 ms	$T_{E}$ = 34.6–155 ms	$T_{E}$ = 2.1–20 ms
		$T_{R}$ = 352–3379 ms	$T_{R}$ = 700–6370 ms	$T_{R}$ = 6000–11 000 ms	$T_{R}$ = 4.9–3285 ms
Dataset22	1.5 T GE	$T_{E}$ = 6.33–12 ms	$T_{E}$ = 99–110 ms	$T_{E}$ = 120–127 ms	$T_{E}$ = 2.56 ms
	and	$T_{R}$ = 360–800 ms	$T_{R}$ = 2680–8480 ms	$T_{R}$ = 6000–8000 ms	$T_{R}$ = 7.96 ms
	1.5 T Philips			$T_{I}$ = 2000 ms	
Dataset24	1.5 T GE	$T_{E}$ = 1.83 ms	$T_{E}$ = 122 ms	$T_{E}$ = 142 ms	$T_{E}$ = 2.18 ms
		$T_{R}$ = 5.98 ms	$T_{R}$ = 4162 ms	$T_{R}$ = 9350 ms	$T_{R}$ = 6.85 ms
				$T_{I}$ = 2200 ms	

### 2.2 Data preprocessing

**FIGURE 2:**
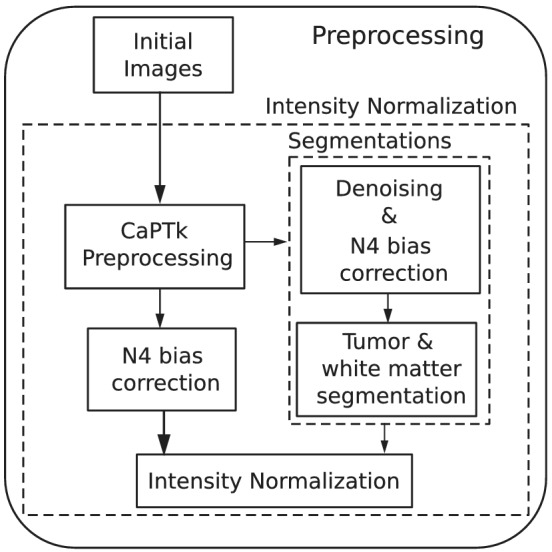
Preprocessing pipeline. Initial images are first co‐registered and skull‐stripped following the CaPTk pipeline. Afterwards, the different contrast images are denoised and bias‐corrected before obtaining the white matter and tumor segmentations. Finally, skull‐stripped images are bias‐corrected and intensity normalized using the segmentations

All weighted images were first co‐registered to 1 mm^3^ isotropic resolution and skull‐stripped^22^ following BraTS preprocessing in CaPTk.^23^ Then, denoising^24^ followed by N4 bias correction^25^ were performed in order to obtain white matter^26^ and tumor segmentations. nNUnet^27^ was utilized to segment the tumor into three distinct regions (ET, non‐enhancing tumor (NET), and edema (ED)) for feature extraction. On the other hand, N4 bias correction^25^ was applied on the skull‐stripped images and these were next normalized by dividing each by the mean intensity of the white matter region contralateral to the tumor. This latter pipeline produces the images input to the RS and to the synthesis procedure. Figure 2 depicts the preprocessing pipeline.

### 2.3 RS for SP of glioblastoma patients

Our RS was trained to classify patients according to the survival criterion (survival >≤ 480 days). The threshold of 480 days (i.e., 16 months) was chosen in order to achieve a balance between group sizes in the test dataset. BraTS2020, TCIA, and Dataset22 (175 patients in total, see Table 1) were used to train the RS. Dataset24 (24 patients) was used for testing in coordination with the synthesis method described in Section 2.4.

Starting from a total of 117 088 handcrafted features extracted from the weighted images, we trained the RS following a nested cross‐validation scheme (outer, 5‐fold; inner, 10‐fold). Feature selection methods^28^, ^29^ were repeated in each outer split to reduce the possible bias produced if training were done on a single cross‐validation split. For each outer split, the model with the lowest Brier loss in the inner split was chosen. Note that five models were selected for the following screening due to the fivefold decision of the outer split. Each of these models was then validated with the validation data corresponding to its outer split. Finally, the model with the best performance, in terms of area under the curve (AUC), was selected. The complete pipeline is outlined in Appendix A.

Using the methodology previously outlined, the best model for each of the three scenarios described below was chosen.

- When the four weighted images (i.e., T1w, T2w, FLAIR, and T1w‐c) were used as input of the RS, the selected model turned out to be an extreme gradient boosting (XGB) with 17 features, two of which are from FLAIR and two from T2w.
- When the channel fed with FLAIR was discarded, the resulting model was a logistic regression (LR) classifier with 16 features.
- When the channel fed with T2w was discarded, a support vector machine (SVM) classifier with 16 features was selected.

Hereinafter, these three models are termed XGB17, LR16, and SVM16, respectively. Features selected for each of the previous models are listed in Supporting Information Tables S4, S5, and S6, respectively.

### 2.4 Synthesis method using a self‐supervised CNN

**FIGURE 3:**
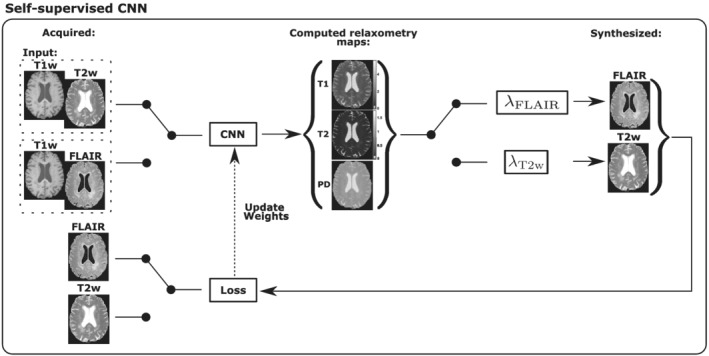
Overview of the self‐supervised CNN. The inputs of the network are T1w and T2w for the synthesis of FLAIR, and T1w and FLAIR for the synthesis of T2w. Note that all the switches change depending on the weighting we want to synthesize. The lambda layers implement the ideal equations indicated in Appendix B

In Reference ^15^ we proposed a synthetic MRI approach for computation of the $T_{1}$, $T_{2}$, and proton density (PD) relaxometry maps and the synthesis of different weighted images from only a pair of inputs. A U‐Net convolutional neural network (CNN) trained with synthetic data was employed. However, some synthesized weightings, such as FLAIR, presented relatively low quality presumably due to the exclusively synthetic training. Thus, if only a few image contrasts are of interest, this issue can be overcome by extending the CNN^15^ into a self‐supervised CNN to be trained with acquired images with the desired contrast. Such an extension has been performed in this work and is graphically represented in Figure 3.

The original CNN was configured with two encoders for the two input weighted images, namely, T1w and T2w or T1w and FLAIR (in the case of synthesizing FLAIR or T2w, respectively). Then, the latent representations of each encoder were fused using a pixel‐wise max function. Finally, we configured three decoders for generation of the $T_{1}$, $T_{2}$, and PD relaxometry maps. In order to extend the CNN into a self‐supervised CNN (see Figure 3) we have included a non‐trainable lambda layer after the decoder's output. This lambda layer implements ideal equations that describe the MR intensity of the output weighted image as a function of the relaxometry maps and the acquisition parameters. These well known equations can be found in Appendix B. The loss function used to train the self‐supervised CNN, named $L_{\text{syn}}$, is computed in the weighted image domain as the average of the mean absolute error (MAE) between each acquired image and its synthesized counterpart. Specifically, let $m^{k}(x)$ denote the intensity value of the k th acquired image at pixel x, defined in some domain $\chi^{k}\subset {\mathbb{R}}^{2}$, and let $m_{\text{syn}}^{k}(x)$ be the synthesized image at that pixel location. Then,

(1) $$\text{MAE}\left(m^{k},m_{\text{syn}}^{k}\right)=\frac{1}{\left|\chi^{k}\right|}\sum_{x\in \chi^{k}}\left|m^{k}(x)-m_{\text{syn}}^{k}(x)\right|$$

with $m^{k}$ and $m_{\text{syn}}^{k}$ vectors that represent the image intensity values of the acquired and synthesized images respectively in all the pixels belonging to domain $\chi^{k}$ with cardinality $\left|\chi^{k}\right|$. Then, the loss function is defined as

(2) $$L_{\text{syn}}=\frac{1}{M}\sum_{k=1}^{M}\text{MAE}\left(m^{k},m_{\text{syn}}^{k}\right),$$

with M the overall number of images entering the average (i.e., the batch size).

Dataset24 (see Table 1) was used to train the self‐supervised CNN following a leave‐one‐out scheme (i.e., a total of 24 models were trained). To train each model, one patient was used for testing and the remaining 23 patients were randomly split between training (18 patients, approximately 80%) and early‐stopping validation (5 patients, approximately 20%).

During training, the loss function was optimized using the Adam algorithm with a learning rate α of 1 × 10^−4^. Further, we empirically fixed the batch size to 32 images. We ran the code using the TensorFlow backend^30^ on a single NVidia GeForce GTX 1070. The total learning took approximately 1 h of computation time for each model, but execution reduces to a few seconds once the network is fully trained.

### 2.5 Experiments

We carried out three test experiments to assess the performance of using synthesized images as input of an RS for SP. In all of them the RS was tested with Dataset24. These experiments are detailed next.

(I) XGB17 was tested with the acquired T1w, T2w, FLAIR, and T1w‐c images as inputs.
(II) XGB17 was tested replacing one of the acquired inputs (FLAIR and T2w, one at a time) by its synthesized version. Therefore, in this experiment three of the inputs of the RS were acquired and one was synthesized. As previously stated, these synthesized images were the test images from the leave‐one‐out scheme of the synthesis method, so no overlap between the training and testing splits occurs in either the synthesis or in the RS.
(III) Models LR16 and SVM16 were tested without considering as input FLAIR or T2w, respectively. Note that the RSs used in this third experiment have been built with only three input channels.

Performance assessment is twofold. On the one hand, we evaluated the quality of the synthesized images. In addition to visual assessment, we also carried out a quantitative analysis using the well known measures^15^ mean squared error (MSE), structural similarity index (SSIM), and peak signal to noise ratio (PSNR). These metrics have been defined within a 3D domain between the synthesized and the acquired images, specifically, within the smallest cube that comprises the foreground of each volume; hence $\chi^{k}$ in Equation (1) is the intersection between this domain and the k th acquired image. Thereafter, the mean and standard deviation values across patients were calculated.

On the other hand, in order to compare the performance of the RS with the different experiment configurations, we computed AUC, accuracy, precision, recall, and $F_{1}$‐score as classifier performance metrics. These metrics were reported as the average value over the two classification classes. Additionally, for the sake of completeness, we analyzed the predicted probabilities of survival obtained at the output of the RS for the pairs Experiments I–II and I–III. To this end, we calculated the $R^{2}$ value, customarily used in linear regression, to measure how predicted probabilities of Experiments II and III deviated from the identity function at abscissae equal to the probabilities of Experiment I. The intra‐class correlation coefficient (ICC)^31^ between these pairs was also measured and boxplots of the probability differences for these pairs were constructed.

## 3 RESULTS

**FIGURE 4:**
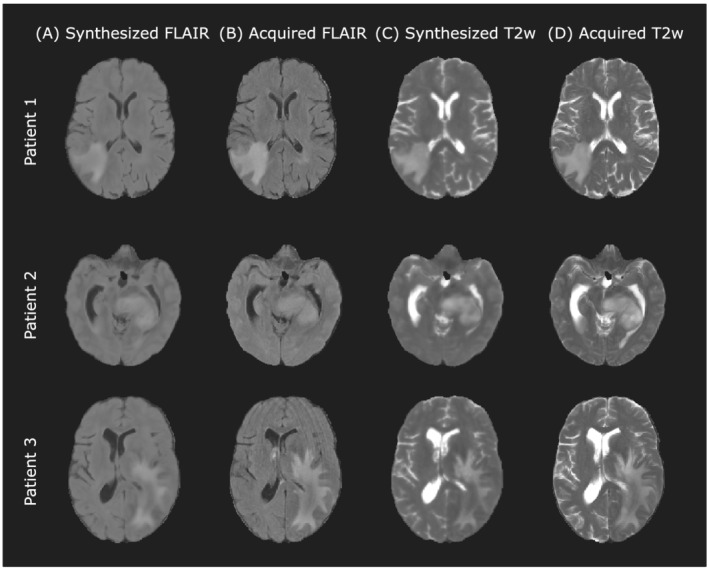
A representative axial slice of the images synthesized by the self‐supervised CNN for different test patients of Dataset24. A, Synthesized FLAIR images. B, Corresponding actually acquired FLAIR images. C, Synthesized T2w images. D, Corresponding actually acquired T2w images

Figure 4 shows a representative slice of the synthesized and the corresponding acquired weighted images for several test glioblastoma patients. Overall, the synthesized images are close to the acquired versions regarding structural information and contrast between tissues in both healthy and pathological regions. In particular, the contours and intensities of the different lesion areas are similar in both. Note that in Patient 3 the synthesized FLAIR image does not suffer from motion artifacts, which are present in the acquired image.

Additionally, Table 3 shows the mean and standard deviation values computed across patients of the synthesis quality metrics between synthesized and acquired images for FLAIR and T2w. SSIM is a value ranging between 0 and 1, and the value 1 is only achievable for two identical images. The high values of PSNR and the low values of MSE show low error between the synthesized and the acquired weighted images for both FLAIR and T2w. All the metrics improve considerably with respect to those obtained by Moya‐Sáez et al^15^ for FLAIR. Note that the comparison of the values obtained for T2w is not representative since this weighted image was input to the CNN in that work.^15^

**TABLE 3:** Synthesis quality metrics used to evaluate the capability to synthesize FLAIR and T2w weighted images. These metrics are the MSE, SSIM, and PSNR. Mean and standard deviation (between parentheses) computed across patients are reported. The metrics were calculated between both the synthesized and the acquired images

	MSE	SSIM	PSNR
FLAIR	0.0163	0.7595	23.7975
	(0.0104)	(0.0474)	(2.0011)
T2w	0.0742	0.7845	25.8934
	(0.0319)	(0.0616)	(1.9032)

**FIGURE 5:**
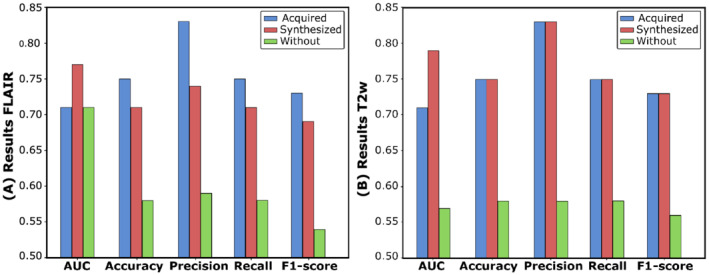
AUC, accuracy, precision, recall, and $F_{1}$‐score of the RS tested with Dataset24 when FLAIR (A) and T2w (B) are acquired, synthesized, or ignored in the whole pipeline

Figure 5 shows the AUC, accuracy, precision, recall, and $F_{1}$‐score achieved for the RS for the three different experiment configurations (Experiments I, II, and III defined in Section 2.5). All the metrics are substantially better in the case of using a synthesized image rather than using a system without such a weighted image for both FLAIR and T2w. The comparison between using an acquired and a synthesized image shows that the performance of the system does not diminish in terms of AUC, and only suffers from a slight degradation in terms of the other performance metrics for FLAIR. Such degradation is not observed with the synthesized T2w.

**FIGURE 6:**
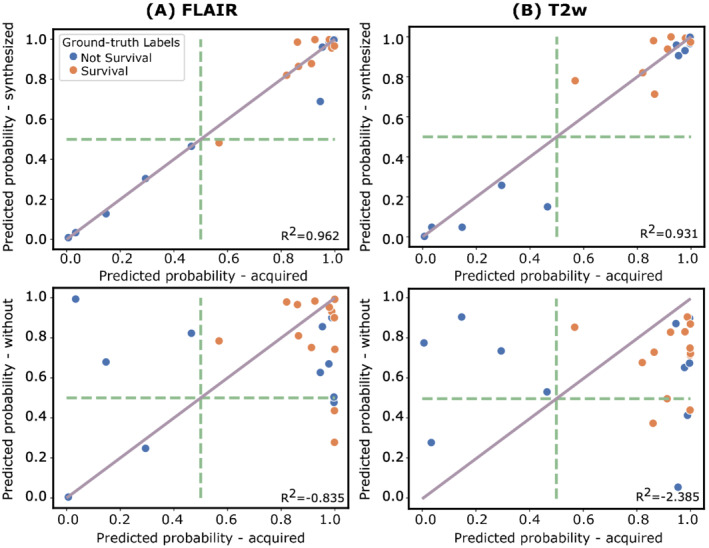
Scatter plots of the predicted probabilities obtained at the output of the RS for the experiment with the acquired versus the synthesized images (top row) and versus an RS trained from scratch without considering this weighted image as input (bottom row). The plots are shown for FLAIR (A) and T2w (B). Dashed lines represent the threshold fixed in the RS to classify survival (>480 days). Each point represents one glioblastoma patient and its color corresponds to the ground‐truth labels. $R^{2}$ values from the identity linear regressions are provided

Figure 6 shows scatter plots of the output predicted probabilities of Experiments I–II and I–III. The ground‐truth labels are also displayed. Additionally, $R^{2}$ values from the identity linear regressions are provided. A better agreement of points in plots in the upper row (Experiments I–II) compared with the plots in the lower row (Experiments I–III) can be observed. This better agreement is also confirmed with the higher values of $R^{2}$ obtained. Additionally, the ICC values measured are 0.983 and 0.292 for FLAIR and 0.964 and 0.027 for T2w for the pairs Experiments I–II and I–III, respectively.

**FIGURE 7:**
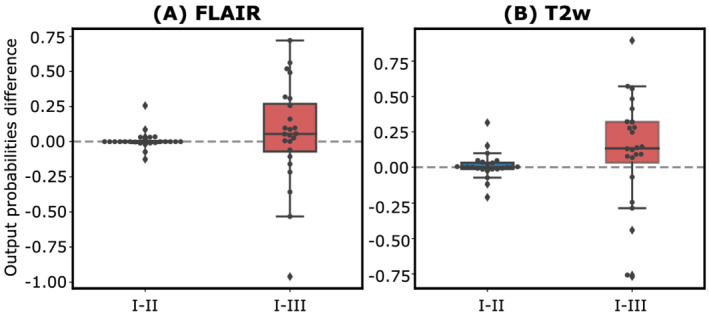
Boxplots of the differences between the probabilities obtained at the output of the RS for Experiments I–II and I–III. Each point represents the probability difference for each glioblastoma patient and the dashed line corresponds to zero difference

Finally, Figure 7 shows boxplots of the probability differences for the pairs Experiments I–II and I–III, for both FLAIR and T2w. As can be seen, the median of the boxplots is closer to zero in Pair I–II than in Pair I–III for both weighted images. A lower interquartile range (IQR) in boxplots of Experiments I–II compared with Experiments I–III can be also observed, together with a median shift from zero in the I–III experiment, an effect which is more prominent for T2w.

## 4 DISCUSSION

In this work, we have thoroughly analyzed the replacement of an actually acquired weighted image with a synthesized version for predicting survival of glioblastoma patients with a completely independent RS. Starting from two acquired weighted images, we synthesized a new weighted image using a CNN‐based method. The RS was trained using as input acquired images only. Then, the system was tested using as input acquired images on one side, and replacing one acquired image with a synthesized image on the other. We also compared performance with a system trained from scratch ignoring this additional channel.

Results show that multicontrast‐demanding quantitative applications, such as radiomics, can be leveraged by synthesized images. Synthesized images may allow widespread usage of these RSs in clinical practice, by retrospectively completing databases with missing modalities and/or replacing artifacted images. Further, these synthesized images have the potential to speed up acquisition protocols by replacing some acquired images with their synthesized versions. In particular, removing FLAIR or T2w from an average brain protocol may reduce the overall scan duration by of the order of 20%, and both of them are artifact‐prone sequences due to their sensitivity to motion. Thus, our results allow us to state that synthetic MRI does add to glioblastoma SP within a radiomics‐based approach. Indeed, the network described in Reference ^15^ is prepared to synthesize more than one contrast, so for protocols with more sequences than those used in this paper more reductions could be potentially achieved.

Our work shows that synthesized weighted images are visually similar to the acquired images in both healthy and pathological tissues. Additionally, the values of image quality metrics prove the agreement between the synthesized and the actually acquired images. The performance achieved with the synthesized images in the RS is not only close to the performance achieved using the acquired images, but also substantially better than using a model trained without this weighting. This is confirmed by the classifier performance metrics (i.e., AUC, accuracy, precision, recall, and $F_{1}$‐score) for both FLAIR and T2w. The $R^{2}$ of the identity linear regression and the ICC values also support this finding. Note that an ICC value above 0.9 is considered excellent.^31^ It is worth noting that the synthesized images input to the XGB17 model improve the AUC compared with acquired images. This might be caused by the implicit filtering undergone during the synthesis procedure. Moreover, accuracy values obtained from the RS are on par with other RSs that rely exclusively on acquired images,^32^ and set the survival threshold for classification, similarly to ourselves, to achieve balance between group sizes.

Our work shows several differences from that described by Islam et al^19^ that are worth highlighting. First, our feature selection procedure turned out to rely on radiomic features mainly based on textures; in contrast, Islam et al^19^ propose a radiogenomic model with 51 features, 43 of which are genetic and 8 radiomic. Most of the latter are morphological, i.e., features that do not depend directly on the image intensity or texture but only indirectly through a segmentation process, and, consequently, they may not be the optimum features to capture the effects of the synthesized images in the classification. On the other hand, in their work Reference ^19^ image synthesis and classification are coupled, since classifiers are trained with synthetic images. Moreover, the results provided there are based on validation data, while our performance figures are calculated from a separate dataset, so we do provide a guarantee to avoid overly optimistic results.

One might argue about the need to synthesize weighted images to feed an RS since relaxometry maps are generated as a previous step to synthesize such weighted images; certainly, the RS could have been designed directly on these maps, and this is a topic in which some other predictions have been properly proposed.^33^ However, we have two reasons for our design choice. First, the glioblastoma databases we have used do not include relaxometry maps. Second, the approach proposed in Moya‐Sáez et al^15^ has been trained with glioblastoma‐free images, both from synthetic data and with a small cohort of real patients. Hence, we needed to extend our original method^15^ to accommodate glioblastoma information. This was achieved by means of a self‐supervised approach trained with weighted images, the ones to which we had access for this type of pathology.

This work has several limitations. The test experiments were carried out on Dataset24, composed of a cohort of 24 glioblastoma patients. We made this design decision because Dataset24 is the only one in which the pulse sequence and the acquisition parameters remained steady across patients, and the self‐supervised synthesis method depends on these parameters. This dependence has the advantage of making the process specific to this parameter setting, so higher synthesis quality can be expected. The downside is the inherent limitation to this particular setting. Nevertheless, the self‐supervised method can be easily extended to accommodate more parameter values for which acquisitions are available. In any case, since 24 patients is not a very large number, experiments on a larger cohort would be advisable to further support our conclusions. Further, a multi‐institutional study could be necessary to analyze the system' generalization capability.

As future work, performing experiments synthesizing post‐contrast weighted images might be of interest from a clinical point of view, in order to avoid the administration of contrast agents to patients. Recently, two such attempts^34^, ^35^ have been reported, although they still have some limitations: first, the lack of versatility, since neither the pulse sequence nor the contrast evolution can be controlled in these methodology; there are also physical limitations due to the fact that contrast‐related information might not be fully embedded in all kinds of pre‐contrast images.^35^ On the other hand, for glioblastoma SP, there is still a wide gap for improvement. New data harmonization strategies, image resolution upgrade, and the combination of other data sources, such as histologic samples, diffusion tensor imaging, or genomics, may better characterize the broad heterogeneity of this disease.

In conclusion, in this work we assessed the performance of an RS when an input actually acquired was replaced with a synthesized version. To this end, we synthesized realistic FLAIR and T2w images in a glioblastoma dataset with a deep learning approach. Furthermore, an RS for SP, which can classify patients into two groups (survival > 480 days and ≤ 480 days) was built. We evaluated the effects of the synthesized weighted images in the RS performance. Results support the utility of using synthesized images to feed an RS for SP of glioblastoma patients.

## Supporting information



nbm4754‐sup‐0001‐SupplementaryMaterial final.pdf

## APPENDIX A RADIOMICS PIPELINE: FEATURE EXTRACTION, FEATURE SELECTION, AND CLASSIFIER TRAINING

### A.1

Different prognostic models have been recently developed for SP of glioblastoma patients. These models utilize a wide range of statistical and machine learning algorithms to analyze heterogeneous data sources and predict patient survival. In this work we propose an RS based on intensity, texture and morphological features extracted from structural MR weighted images used to test the quantitative performance of the synthesized images.

### Feature extraction

A total of five ROIs were derived from the tumor segmentation. Three of them are directly defined in the segmentation—ET, NET, ED—and two more are constructed from them—tumor core (TC), which is the union of ET and NET, and whole tumor (WT), the union of the three initial regions. Moreover, 10 different filters are applied to the images. Wavelet filtering is applied using one decomposition level of the db2 Daubechies orthogonal wavelet, yielding eight decompositions; these are all possible combinations of applying either a high or a low pass filter in each of the three dimensions. Also, two Laplacian of Gaussian filters are applied to emphasize areas of gray level change; this type of filter is controlled by the parameter sigma, which defines how coarse the emphasized texture should be. Two sigma values have been used (2 and 5). These features have shown improvements in the final prediction result.^36^

From the previously mentioned five ROIs, four weightings, and 11 images (both the original and the 10 filtered images), a total of 117 088 features were extracted. MATLAB was used to define and extract these features. In particular, for texture features, the package presented by Dancheva et al,^37^ which is IBSI compliant,^38^ was employed. These features are defined as follows.

- Volume features (six features): volume of WT, TC, each region (ET, NET, and ED), and volume of the brain.
- Volume ratios (seven features): ratio of WT and the brain, ratio of ET and WT, ratio of NET and WT, ratio of ED and WT, ratio of ET and NET, ratio of ET and ED, and ratio of NET and ED.
- Morphological features (34 features): the percentage of the TC inside the cerebellum, brain stem, basal ganglia, and the parietal, occipital, frontal, and temporal lobes. These measurements are obtained by segmenting the SRI‐24 atlas with FreeSurfer,^39^ fusing the FreeSurfer regions to create the preceding anatomical areas and taking advantage of the rigid registration of the different cases to the SRI‐24 atlas to measure the presence of the tumor in those regions (see Supporting Information Figure S1). These measurements are also calculated for the left and right hemispheres. Furthermore, the centroid of the tumor is determined and measurements of compactness, sphericity, the ratio volume to surface of the tumor, the TC surface area, and another sphericity measurement defined by Pérez‐Beteta et al^40^ are computed.
- First order, histogram‐based, and texture features ( 68×4×5×11 features): 68 features are calculated over the whole ROIs. Calculated features are named in Supporting Information Table S2.
- Extracted features from feature maps ( 58×8×4×5×11 features): Each feature from Supporting Information Table S2 is computed on a 3×3×3 pixel block within each ROI to create different feature maps (see Supporting Information Figure S2). In Supporting Information Table S2, the 20 histogram‐based features are replaced by 10 features derived from the frequency and probability values of the five‐bin frequency and probability histograms constructed in the 3×3×3 blocks. As a consequence, the total number of features from which feature maps are derived is reduced from 68 to 58. Following this, the eight first‐order features are calculated on these maps, yielding feature distribution measures along the feature maps. This method, which involves creating feature maps, may be able to improve the tumor heterogeneity characterization, which is a critical aspect of glioblastoma.^41^

Patient age is added to the group of the first three categories defined above, so this group consists of 6+7+34+1=48 features. The total number of features can be calculated as the following summation:

(58×8+68)×4×5×11+48=117.088

with 4 the number of weighted images, 5 the number of ROIs and 11 the sum of the 10 filtered images—8 wavelet filters and 2 Laplacian of Gaussian filters—plus the original image.

### Feature selection and model training

The RS was trained following a 10‐fold‐within‐5‐fold nested cross‐validation procedure. The method employed is the following.

- The total data was first divided into a training (175 patients) and a testing set (24 patients). Outliers, defined as feature values above the 99th percentile or below the first percentile, were clipped and features were normalized by removing the mean and scaling to unit variance using the training set as reference.
- Five splits are performed over the training data (140 patients outer training and 35 patients outer validation). On each of these splits feature selection is executed.
- The outer training split is divided 10 times (125 patients for inner training, 15 patients for inner validation), and on these splits the training of five different machine learning models is carried out with the features selected in the outer split. Brier loss is calculated for each classifier and the best performer is chosen for each outer split.
- Each of the five models selected in the previous step is trained on its outer training data (140 patients) and validated on its outer validation data (35 patients). The model with the highest AUC on its validation data is the selected model.
- The selected model is finally trained on the training data (175 patients) and tested on the testing data set (24 patients).

Feature selection in each outer loop is done following a three‐step process.

1. First, the Spearman correlation between each feature and survival in days is calculated, and features that show a statistically significant correlation ( p<0.05) are retained. In addition, the Spearman correlation matrix between the statistically significant features is calculated, and the correlation values are used to eliminate those that are correlated with each other (correlation factor > 0.66), keeping the feature with the lowest p‐value.
2. Second, the TuRF method was applied.^28^ TuRF addresses feature interaction iteratively utilizing the Relief method deriving feature statistics based on nearest neighbors. Features with the lowest scores are recursively eliminated. The number of features is narrowed to the top 100 with the best TuRF weights. In addition, age and 19 morphological and position information features, detailed in Supporting Information Table S3, are reincorporated at this point. This type of feature has been demonstrated as highly reproducible and able to improve model performance.^42^
3. Finally, an information measure, maximum relevance–minimum redundancy (mRMR),^29^ was utilized to obtain the final subset. This metric generates a ranking of feature groups by iterating in the cardinality of the groups. Specifically, mRMR is set to create 50 sets with cardinality one, 50 with cardinality two and so forth until cardinality 30. Each feature set that mRMR generates is tested on different classifiers in each outer split and, for the sets of the same cardinality, the one with the lowest Brier loss (in the inner splits) is selected. Calculating AUC with the validation data in the outer split, we select the best classifier–cardinality pair of the feature set. The effective cardinality of feature sets, however, has been set smaller by applying the “one in 10 rule,”^43^ so we have feature sets with cardinality less than or equal to 17. Supporting Information Figure S3 shows that only slight improvements in AUC are obtained when increasing the number of features beyond this threshold.

Four different models available in the scikit‐learn library,^44^ namely, naive Gaussian Bayes, LR, random forest, and SVM, as well as the XGB^45^ model, were employed. The model and features selected for Experiments I and II are defined in Supporting Information Table S4. The model selected in this case was XGB. Feature importance, shown in Supporting Information Figure S4, is calculated as the average gain of the splits in which the feature appears for all trees of the model.^45^ Furthermore, the features selected for the Experiment III, both without FLAIR and without T2w, are listed in Supporting Information Tables S5 and S6. For these cases the models used were LR and SVM, respectively. Models' hyperparameters during training are given in Supporting Information Table S7.

## APPENDIX B IDEAL SYNTHESIS EQUATIONS

### B.1

Many MR weighted images can be analytically synthesized using the well known equations that describe image intensity as a function of acquisition parameters, such as echo time ( $T_{E}$), repetition time ( $T_{R}$), and inversion time ( $T_{I}$), in relation to the involved relaxometry maps. In this work, based on the $T_{1}$, $T_{2}$, and PD relaxometry maps, we synthesized weighted images corresponding to the sequences spin echo (SE) for synthesis of the T2w image, and inversion recovery spin echo (IR‐SE) for synthesis of the FLAIR image, with respective equations

(B1)
$$m_{\text{SE}}(x)=\text{PD}(x)\left[1-2e^{-(T_{R}-T_{E}/2)/T_{1}(x)}+e^{-T_{R}/T_{1}(x)}\right]e^{-T_{E}/T_{2}(x)}$$

(B2)
$$m_{\text{IR‐SE}}(x)=\text{PD}(x)\left[1-2e^{-T_{I}/T_{1}(x)}+2e^{-(T_{R}-T_{E}/2)/T_{1}(x)}-e^{-T_{R}/T_{1}(x)}\right]e^{-T_{E}/T_{2}(x)}$$

where m(x) the signal intensity of the corresponding weighted image at voxel location x defined on some domain X. The acquisition parameters used in this work for each of the equations are given in Table 2.
