# Supplementary material for: Synthetic MRI improves radiomics‐based glioblastoma survival prediction
Source: NMR Biomed. 2022 May 21;35(9):e4754. doi: 10.1002/nbm.4754 (PMC9542221; doi:10.1002/nbm.4754)
Supplement: Supplementary file 1 — nbm4754‐sup‐0001‐SupplementaryMaterial final.pdf [file NBM-35-e4754-s001.pdf]

Supplementary Material

February 24, 2022

Supplementary Material Figure S1: Tumor-core (TC) presence in the different lobes of the brain for a representative training patient of *BraTS2020*. As seen in the image, the tumor is mainly located in the posterior part of the parietal and temporal left lobe. Features extracted are the percentage of the tumor in each region.

Supplementary Material Figure S2: Feature map standard deviation calculated over a block of 3x3x3 voxels in a representative training patient of the *BraTS2020* dataset.

Supplementary Material Figure S3: AUC change when modifying the number of features for each outer split in the nested cross-validation scheme. The models evaluated are the best performers by AUC.

Supplementary Material Figure S4: Feature importance for the model selected. Importance is computed as the mean and standard deviation of accumulation of the impurity decrease (MDI) within each tree. Feature numeration follows Supplementary Material Table S4. FLAIR features are 9 and 11. T2w features are 10 and 14.

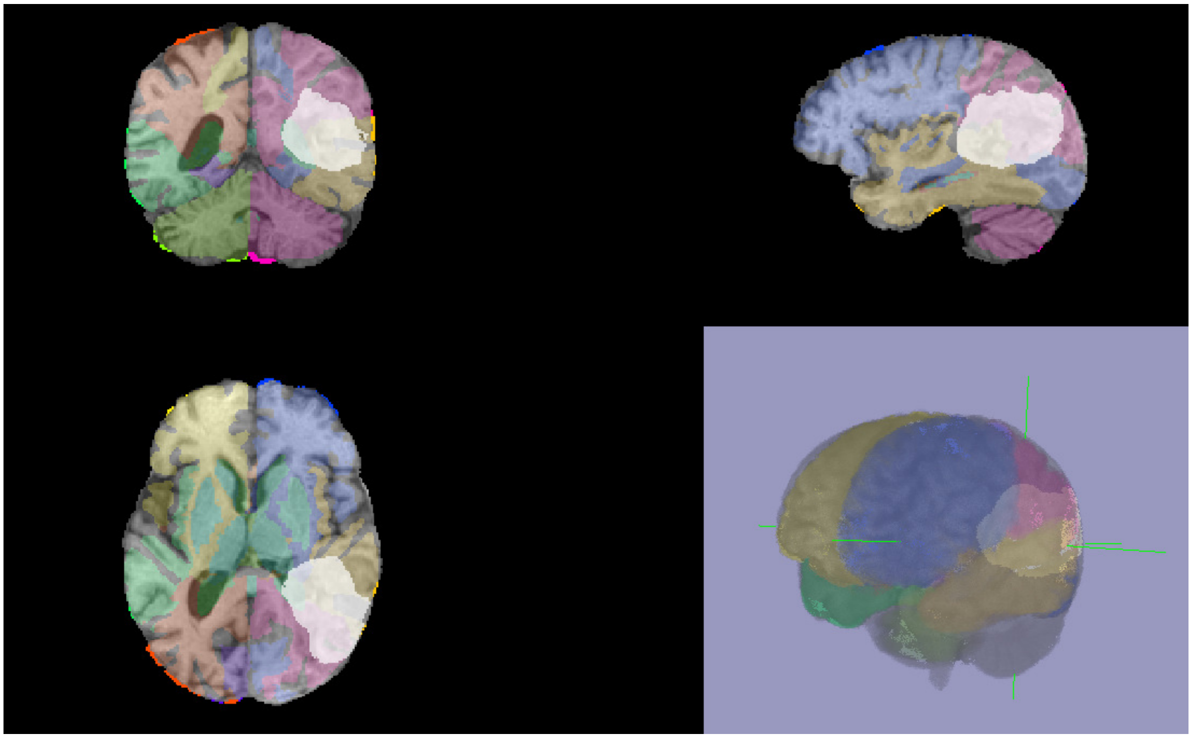

Supplementary Material Figure S1: Tumor-core (TC) presence in the different lobes of the brain for a representative training patient of *BraTS2020*. As seen in the image, the tumor is mainly located in the posterior part of the parietal and temporal left lobe. Features extracted are the percentage of the tumor in each region.

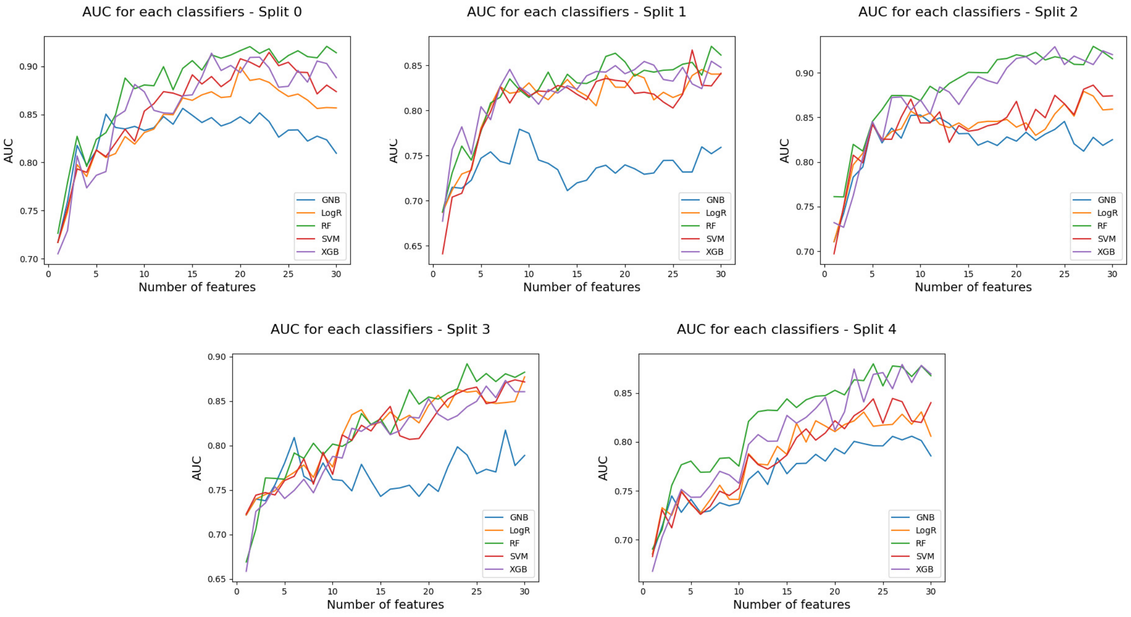

Supplementary Material Figure S2: Feature map standard deviation calculated over a block of 3x3x3 voxels in a representative training patient of the *BraTS2020* dataset.

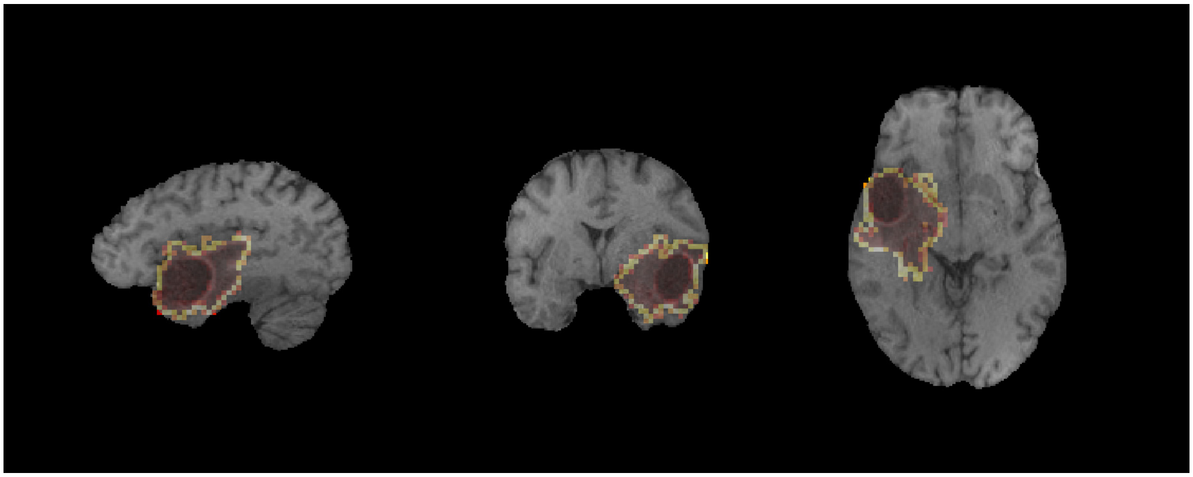

Supplementary Material Figure S3: AUC change when modifying the number of features for each outer split in the nested cross-validation scheme. The models evaluated are the best performers by AUC.

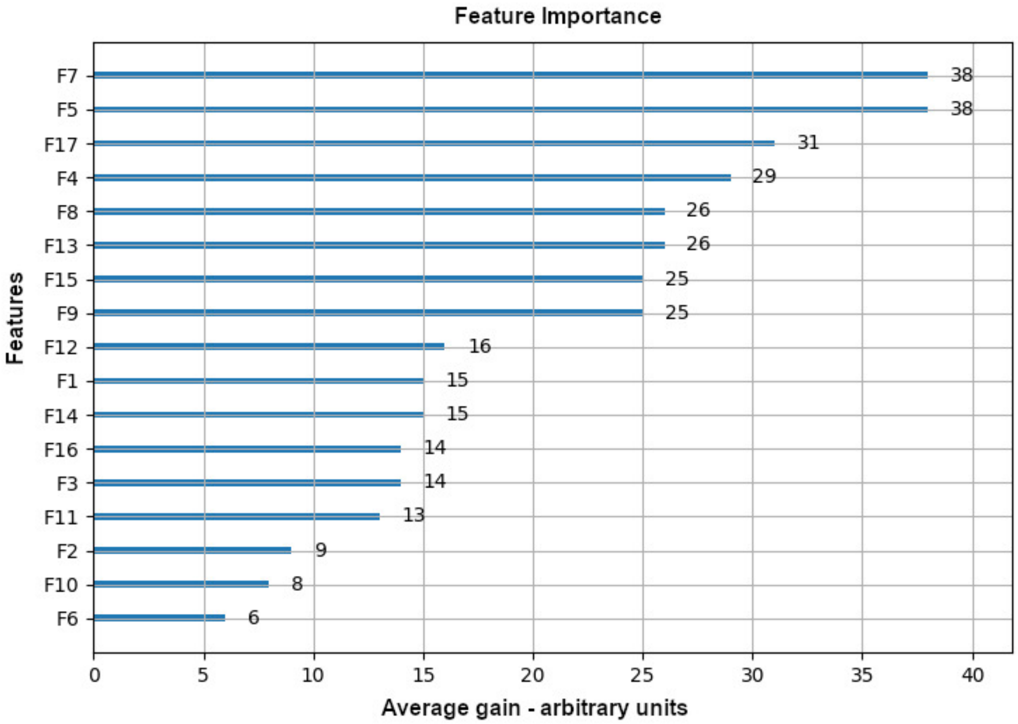

Supplementary Material Figure S4: Feature importance for the model selected. Importance is computed as the mean and standard deviation of accumulation of the impurity decrease (MDI) within each tree. Feature numeration follows Supplementary Material Table S4. FLAIR features are 9 and 11. T2w features are 10 and 14.

Supplementary Material Table S1: Cases selected from the public datasets *BraTS2020* and Ivy-GAP, CPTAC, and TCGA available through *TCIA*.

Dataset	Cases
BraTS2020	001, 002, 003, 004, 005, 006, 007, 009, 010, 012, 013, 014, 015, 016, 017, 018, 019, 021, 022, 023, 024, 028, 033, 034, 036, 037, 039, 042, 048, 049, 053, 054, 056, 057, 058, 059, 060, 061, 062, 064, 065, 066, 067, 068, 069, 070, 071, 072, 074, 076, 077, 078, 079, 080, 082, 083, 084, 086, 089, 090, 091, 093, 095, 096, 097, 098, 099, 100, 101, 102, 103, 104, 106, 107, 108, 110, 111, 112, 113, 114, 115, 116, 117, 118, 119, 120, 121, 122, 123, 124, 125, 126, 127, 128, 129, 145, 147, 151, 152, 153, 154, 157, 337, 339, 341, 342, 345, 346, 347, 351, 356, 357, 358, 359, 360, 363, 366, 368, 369
Ivy-GAP	W1, W2, W5, W6, W8, W10, W11, W13, W19, W20, W22, W29, W32, W34, W35, W40, W43, W48, W54,
CPTAC	C3L_00278, C3L_00424, C3L_00528, C3L_00591, C3L_00677, C3L_02041, C3L_02504, C3L_03266, C3L_01505, C3L_00349, C3L_01327, C3L_02465, C3L_03727
TCGA	TCGA-14-1794, TCGA-14-1829

Supplementary Material Table S2: First order, histogram-based, and texture features. Texture features are extracted using 4 different texture metrics, neighbouring gray tone difference matrix (NGTDM), gray level run length matrix (GLRLM), gray level co-occurrence matrix (GLCM), and gray level size zone matrix (GLSZM).

Feature type	Feature	Nº
**First order**	Kurtosis, skewness, variance, standard deviation, median, mean minimum, and maximum	8
**Histogram-based**	Each bin of a 20 bins histogram	20
**NGTDM**	Coarseness, Contrast, Bussyness, Complexity, Strength	5
**GLRLM**	GLN, GLV, HGRE, LGRE, LRE, LRHGE, LRLGE, RLN, RLV, RP, SRE, SRHGE, SRLGE	13
**GLCM**	Energy, Contrast, Entropy, Homogeneity, Correlation, Variance, SumAverage, Auto Correlation, Dissimilarity	9
**GLSZM**	SZE, LZE, GLN, ZSN, ZP, LGZE, HGZE, SZLGE, SZHGE, LZLGE, LZHGE, GLV, ZLB	13

Supplementary Material Table S3: Features reintroduced in feature selection step 2.

Nº	Feature	Feature definition
**1**	Age	Age
**2**	rNET_ED	Volume ratio non-enhancing and tumor edema
**3**	rET_NET	Volume ratio enhancing tumor and non-enhancing tumor
**4**	rET_ED	Volume ratio enhancing tumor and edema
**5**	rNET_ED	Volume ratio non-enhancing tumor and edema
**6**	rED_WT	Volume ratio edema and whole tumor
**7**	rNET_WT	Volume ratio non-enhancing tumor and whole tumor
**8**	rET_WT	Volume ratio enhancing tumor and whole tumor
**9**	rNET_WT	Volume ratio non-enhancing tumor and whole tumor
**10**	rWT_B	Volume ratio whole tumor and brain
**11**	Morph_VolTumBasGang	Percentage of the tumor core volume in the basal ganglia measured by the rigid registration of the patients image data on the Freesurfer Segmentation of the SRI-24 atlas
**12**	Morph_mayAxisLegth	Mayor axis length of the tumor core
**13**	Morph_leastAxisLegth	Least axis length of the tumor core
**14**	Morph_coreArea	Area of the tumor core segmentation
**15**	Morph_sphericity	Sphericity calculated as $\frac{(36\times\pi\times coreVol^2)^{\frac{1}{3}}}{Morph\_coreArea}$
**16**	Morph_comp1	Compactness calculated as $\frac{coreVol}{\sqrt{(\pi)}\times\sqrt{(Morph\_coreArea^3)}}$
**17**	Morph_PB_Sr	Sphericity as specified in (**?**) $6\times\sqrt{\pi}\times\frac{coreVol}{\sqrt{(Morph_coreArea^3)}}$
**18**	WT_cores	Number of WT focal points in the brain
**19**	TC_cores	Number of TC focal points in the brain
**20**	NET_cores	Number of non-enhancigng tumor focal points in the brain

Supplementary Material Table S4: Features selected for the model with four weighted images as input. The model selected in that case was extreme gradient boosting.

Nº	Weighted image	ROI	Filter	Feature Map	Feature
**1**	—	—	—	—	TC volume in basal ganglia
**2**	T1w	WT	Wavelet HLH	min	Variance
**3**	T1w-c	ET	Wavelet HHH	—	2nd bin 20 bins histogram
**4**	T1w	NET	LoG F2	GLSZM LZLGE	Variance
**5**	T1w-c	NET	Wavelet HHL	GLCM Homogeneity	Kurtosis
**6**	T1w	NET	Wavelet LLH	—	5th bin 20 bins histogram
**7**	T1w-c	TC	LoG F2	GLSZM LZHGE	Skewness
**8**	T1w	ED	Wavelet LHL	min	Stanadard Deviation
**9**	FLAIR	ET	Wavelet HLL	GLSZM SZHGE	Min
**10**	T2w	ET	Wavelet HLH	GLSZM ZSV	Mean
**11**	FLAIR	TC	Wavelet HLH	GLCM Dissimilarity	Skewness
**12**	T1w	ED	—	—	4th bin 20 bins histogram
**13**	T1w	WT	Wavelet LLH	GLRLM LGRE	Mean
**14**	T2w	ED	—	GLCM Contrast	Max
**15**	T1w	ET	LoG F2	GLRLM RLN	Skewness
**16**	T1w	ED	LoG F2	—	Skewness
**17**	T1w	ET	Wavelet HLH	NGTDM Busyness	Median

Supplementary Material Table S5: Features selected for the model with only three weighted images as inputs (without FLAIR). The model selected in that case was logistic regression.

**Nº**	**Weighted image**	**ROI**	**Filter**	**Feature Map**	**Feature**
**1**	T1w-c	ED	Wavelet LLL	Median	Mean
**2**	T1w	ET	Wavelet HLH	GLSZM SZE	Kurtosis
**3**	T1w	NET	Wavelet LLH	Min	Skewness
**4**	T1w-c	ED	Wavelet HLL	—	17th bin 20 bins histogram
**5**	T2w	ED	Wavelet HLH	GLSZM LZHGE	Variance
**6**	—	—	—	—	Volume ratio between ET and WT
**7**	T1w	ED	Wavelet HLL	GLSZM SZLGE	Mean
**8**	T1w-c	ED	Wavelet LLL	NGTDM Complexity	Standard Deviation
**9**	T1w-c	TC	—	GLSZM SZLGE	Skewness
**10**	T1w	WT	Wavelet LLH	GLRLM LRLGE	Skewness
**11**	T1w-c	ED	Wavelet HLL	GLRLM SRLGE	Min
**12**	T1w	TC	Wavelet HHL	Skewness	Median
**13**	T2w	TC	—	GLCM Dissimilarity	Max
**14**	T1w	ET	Wavelet LLL	Variance	Mean
**15**	T1w	TC	Wavelet LLH	GLSZM	Min
**16**	T1w-c	NET	Wavelet HHL	GLCM Homogeneity	Kurtosis

Supplementary Material Table S6: Features selected for the model with only three weighted images as input (without T2w). The model selected in that case was support vector machine.

**Nº**	**Weighted image**	**ROI**	**Filter**	**Feature Map**	**Feature**
**1**	FLAIR	TC	Wavelet HHL	GLSZM GLV	min
**2**	FLAIR	WT	Wavelet HLL	—	GLSZM SZHGE
**3**	FLAIR	ED	—	GLCM Contrast	Skewness
**4**	—	—	—	—	Age
**5**	T1w	ED	LoG F2	—	15th bin 20 bins histogram
**6**	FLAIR	NET	Wavelet HLL	GLRLM LGRE	Skewness
**7**	T1w	TC	Wavelet LLH	—	GLSZM SZLGE
**8**	FLAIR	WT	Wavelet LHL	Min	Kurtosis
**9**	T1w	TC	—	GLRLM LRHGE	Standard deviation
**10**	T1w	ED	Wavelet LLL	—	3rd bin 20 bins histogram
**11**	—	—	—	—	NET focal points
**12**	T1w	ED	Wavelet LHL	Mean	Median
**13**	FLAIR	WT	Wavelet HHH	—	GLSZM LGZE
**14**	FLAIR	ET	Wavelet HHL	GLCM SumAverage	Min
**15**	T1w	WT	Wavelet LHH	GLRLM LRHGE	Median
**16**	T1w	ET	LoG F2	—	GLRLM HGRE

Supplementary Material Table S7: Models' hyperparameters.

Model	Hyperparameters
Logistic Regression	penalty='l2', *, dual=False, tol=0.0001, C=1.0, fit_intercept=True, intercept_scaling=1, solver='lbfgs', max_iter=100, multi_class='auto', verbose=0, warm_start=False,
Naive Gaussian Bayes	*, priors=None, var_smoothing=1e-09
Random Forrest	n_estimators=500, *, criterion='entropy', max_depth=4 min_samples_split=2, min_samples_leaf=1, min_weight_fraction_leaf=0.0, max_features='auto', min_impurity_decrease=0.0, bootstrap=True, oob_score=False, n_jobs=None, verbose=0, warm_start=False, ccp_alpha=0.0,
Support Vector Machine	*, C=1.0, kernel='linear', degree=3, gamma='scale', coef0=0.0, shrinking=True, probability=False, tol=0.0001, cache_size=200, verbose=False, max_iter=- 1, decision_function_shape='ovr', break_ties=False
Extreme Gradient Boosting	objective: 'binary:logistic', use_label_encoder: False, importance_type: 'gain', n_estimators: 100, verbosity: 0,
